# Degradation Assessment of Poplar Shelterbelts in the Kubuqi Desert Using an Entropy Weight–TOPSIS–RSR Model

**DOI:** 10.3390/plants15121874

**Published:** 2026-06-17

**Authors:** Xue Chen, Haibing Wang, Jin Ni, Xinghua Zhao, Enhe Mengde, Xuan Chen, Hejun Zuo

**Affiliations:** 1 State Key Laboratory of Water Engineering Ecology and Environment in Arid Area, Inner Mongolia Agricultural University, Hohhot 010018, China; chenxue0107@126.com (X.C.);; 2 Inner Mongolia Key Laboratory of Aeolian Physics and Desertification Control Engineering, College of Desert Control Science and Engineering, Inner Mongolia Agricultural University, Hohhot 010018, China; 3 Inner Mongolia Hanggin Desert Ecosystem Positioning Research Station, Ordos 017400, China

**Keywords:** poplar shelterbelts, degradation assessment, grade-specific management

## Abstract

Artificial shelterbelts in arid and semi-arid regions play a key role in controlling land degradation, regulating wind erosion, and maintaining ecological security. However, their long-term protective effectiveness increasingly depends on accurate degradation diagnosis and targeted management of aging and degraded stands. This study developed a comprehensive health assessment and degradation grading framework for poplar shelterbelts in the Kubuqi Desert, northern China, using an indicator system covering stand structure, community structure, soil conditions, health risks, and external disturbances. Indicator weights were determined using the entropy weight method, and degradation grades were classified by combining the technique for order preference by similarity to ideal solution (TOPSIS) model with the rank-sum ratio (RSR)–Probit method. The results showed that soil conditions and stand structure were the dominant dimensions distinguishing degradation status, with weights of 50.98% and 25.30%, respectively. Grade I, Grade II, Grade III, and Grade IV stands accounted for 21.88%, 25.00%, 34.38%, and 18.75% of the plots, respectively, indicating that lightly and moderately degraded stands were predominant. Degradation grades were also associated with changes in understory cover and surface soil nutrients, especially decreases in soil organic matter and alkali-hydrolyzable nitrogen. Based on these results, grade-specific management strategies were proposed, including conservation and maintenance, density regulation, assisted restoration, and near-natural transformation. This framework provides a practical basis for diagnosing degradation status and guiding the renewal and management of aging shelterbelts in arid sandy regions.

## 1. Introduction

Global arid and semi-arid regions are among the areas most seriously affected by land degradation, wind erosion, and declining ecosystem stability, and they are also key regions for ecological restoration and desertification control worldwide [1,2,3]. To mitigate wind–sand hazards, restore degraded land, and maintain regional ecological security, artificial shelterbelts and sand-fixing vegetation systems have been widely established in many arid regions. These systems play important roles in reducing near-surface wind speed, controlling wind erosion, improving habitat conditions, and protecting farmland and infrastructure [4,5]. Similar large-scale restoration efforts have also been implemented in other dryland and water-limited regions. For example, the African Great Green Wall for the Sahara and the Sahel has evolved from a tree-planting belt into a broader landscape restoration initiative aimed at combating land degradation and enhancing social–ecological resilience [6]. In the Mediterranean region, land degradation assessment and forest landscape restoration have also received increasing attention because forests and grazing lands are strongly affected by drought, fire, overgrazing, and land-use change [7]. These international experiences indicate that degradation diagnosis and adaptive management of restoration vegetation are common challenges in dryland and seasonally dry regions worldwide.

In the arid and semi-arid aeolian sandy regions of northern China, large areas of poplar-dominated shelterbelts have been established over the past few decades and have become an important component of windbreak and sand-fixation systems as well as regional ecological barrier construction [8]. However, under conditions of low precipitation, intense evapotranspiration, stand aging, relatively high planting density, and insufficient management, some poplar shelterbelts have gradually shown degradation symptoms, including growth decline, canopy thinning, reduced survival rate, increased mortality, understory vegetation expansion, and changes in soil nutrients. These changes can weaken their protective function and ecological stability [9,10,11]. Recent climate projections further indicate that drought risk and extreme climate events may intensify in China under future warming scenarios, especially in arid and semi-arid regions where warming-driven evapotranspiration can increase vegetation water stress [12,13]. Therefore, against the background of increasing drought stress and land degradation in arid regions, establishing an evaluation method that can accurately identify the degradation status of poplar shelterbelts and support grade-specific management is an important basis for the renewal and rehabilitation of shelterbelts in arid and semi-arid regions [14,15].

Forest health assessment emphasizes the capacity of forest ecosystems to maintain structural stability, functional integrity, and resistance to external disturbances over a given time scale [16,17]. Previous studies have developed forest health or forest condition assessment index systems based on stand structure, soil conditions, biodiversity, and ecological functions. Common indicators include tree height, diameter at breast height (DBH), stand density, canopy closure, mortality rate, soil physicochemical properties, and understory community composition [18,19]. However, most existing studies have focused on natural forests, commercial forests, or urban forests, whereas less attention has been paid to artificial poplar shelterbelts in arid sandy regions, whose primary functions are windbreak and sand fixation [20,21]. For these shelterbelts, degradation is not reflected by changes in a single structural indicator alone, but results from the combined effects of stand structure, understory communities, surface soil conditions, health risks, and external disturbances [9,13,22]. Therefore, it is necessary to develop a health assessment and degradation indicator system that can integrate multidimensional information.

In multi-criteria evaluation, the determination of indicator weights and the selection of a comprehensive evaluation model directly affect the reliability of evaluation results [23,24]. Subjective weighting methods can reflect management experience but are susceptible to human bias, whereas objective weighting methods determine weights based on variations among indicator data, thereby helping to reduce subjective interference [25]. The entropy weight method can reflect the information contribution of different indicators, while the TOPSIS model can convert multi-indicator information into a continuous comprehensive evaluation index. These methods have been widely applied in ecological quality, environmental health, and vulnerability assessments [26,27]. However, continuous health scores alone are still insufficient for direct use in management practice, as shelterbelt management usually requires explicit diagnostic grades, such as non-degraded, lightly degraded, moderately degraded, and severely degraded [28]. Therefore, integrating objective weighting, comprehensive index calculation, and degradation classification is key to improving the management applicability of evaluation results.

The RSR–Probit method can classify continuous comprehensive scores into discrete grades based on rank information and probability distribution characteristics, thereby providing a statistical basis for degradation classification [27,29]. Combining the entropy weight method, TOPSIS model, and RSR–Probit method can establish a complete workflow including indicator system construction, objective weighting, comprehensive index calculation, and degradation grade classification. Further integration with understory community and surface soil responses can be used to examine whether the degradation grades have ecological explanatory power [30,31]. By linking different degradation grades with corresponding management measures, evaluation results can be transformed from statistical rankings into management-oriented information, providing a basis for the classified restoration and fine-scale management of degraded shelterbelts [28,32].

Accordingly, this study focuses on poplar shelterbelts in the Kubuqi Desert, a sandy region of northern China. We constructed a comprehensive evaluation index system covering stand structure, community structure, soil conditions, health risks, and external disturbances. We used the entropy weight method to determine objective weights, applied the TOPSIS model to calculate the comprehensive health index (Ci), and combined it with the RSR–Probit method to classify degradation grades. Based on the ecological characteristics of poplar shelterbelts in arid sandy regions, we proposed two hypotheses: (1) soil conditions and stand structure are the dominant dimensions distinguishing the degradation status of poplar shelterbelts; and (2) degradation grades derived from the comprehensive evaluation system correspond to measurable differences in understory community characteristics and surface soil properties. To test these hypotheses, we further examined the ecological response differences among degradation grades using indicators of understory communities and surface soil, and proposed grade-specific management strategies for different degradation grades. This study aims to establish a comprehensive health evaluation and grade-based diagnostic method for poplar shelterbelts that is calculable, classifiable, interpretable, and management-oriented, thereby providing methodological support for the renewal and rehabilitation of degraded shelterbelts in sandy regions.

## 2. Materials and Methods

### 2.1. Overview of the Study Area

**Figure 1:**
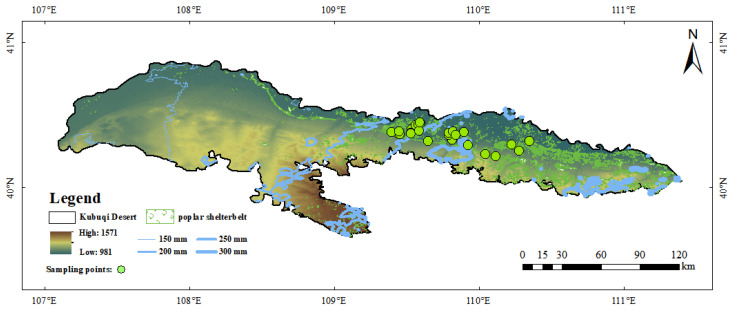
Overview of the study area and distribution of sampling sites.

The study area is located in the Kubuqi Desert and its surrounding shelterbelt construction areas in the northern Ordos Plateau (Figure 1). Situated in the central part of the northern China aeolian sandy belt, it represents a typical arid–semi-arid transition zone. The region is characterized by low precipitation and strong evaporation, with rainfall mainly concentrated from June to September. Frequent wind–sand activity occurs in spring, and the dominant soil type is aeolian sandy soil. Common plant communities include *Caragana korshinskii*, *Artemisia ordosica*, *Salix psammophila*, and *Calligonum mongolicum*. Long-term desertification control and ecological restoration projects have shaped an artificial vegetation pattern dominated by poplar shelterbelts and shrub sand-fixation plantations. Poplar shelterbelts play an important role in windbreak and sand fixation, as well as in protecting roads and agro-pastoral ecotones. However, some stands have shown varying degrees of degradation due to stand aging, relatively high planting density, insufficient management, and external disturbances. Therefore, this region is suitable for conducting comprehensive health assessment and grade-based diagnosis of poplar shelterbelts.

### 2.2. Plot Establishment and Data Collection

Typical poplar shelterbelt plots in the Kubuqi Desert were selected as the study sites. A total of 32 plots were retained for health assessment and degradation grading analysis. The 32 plots were selected to represent the main variation in stand conditions of poplar shelterbelts in the study area and to cover the full degradation gradient from non-degraded to severely degraded stands. During plot selection, stand structure, canopy condition, survival status, dead branch proportion, disturbance intensity, and field accessibility were considered. All field measurements and sampling, including stand structure investigation, understory vegetation survey, and surface soil sampling, were conducted from June to August 2025 during the growing season. The same field investigation and sampling procedures were applied across all plots to reduce the potential influence of seasonal variation on understory cover, soil moisture, and available soil nutrient measurements. Following the plot layout method proposed in previous studies [33], each plot measured 20 m × 20 m. Within each plot, stand structure indicators of poplar shelterbelts were surveyed, including canopy closure, survival rate, mortality rate, tree height, and planting density. Meanwhile, health risks and external disturbance indicators, such as the proportion of dead branches, pest and disease occurrence, and grazing disturbance, were recorded.

In each plot, four 1 m × 1 m herbaceous quadrats were established from the plot center in four directions to investigate community structure indicators, including understory vegetation cover and species richness. Surface soil samples were collected from the 0–20 cm soil layer to determine soil condition indicators, including soil moisture content, bulk density, organic matter, available phosphorus, available potassium, alkali-hydrolyzable nitrogen, pH, and electrical conductivity. These data were used to construct a comprehensive health evaluation and degradation grading index system for poplar shelterbelts.

### 2.3. Development of the Indicator System

Drawing on previous experience in constructing evaluation systems for forest health, forest condition, and ecological health [34,35], this study followed the principles of scientific validity, representativeness, data availability, and comparability to develop a health evaluation and degradation indicator system for poplar shelterbelts. The system included 18 indicators across five major categories: stand structure, community structure, soil conditions, health risks, and external disturbances. The ecological significance of each indicator category is as follows: stand structure indicators, including canopy closure, survival rate, mortality rate, average tree height, and planting density, reflect stand growth status and structural stability; community structure indicators, including herbaceous cover and species richness index, reflect the competitive pattern of understory vegetation and community development status; soil condition indicators, including surface soil moisture content, bulk density, organic matter, available phosphorus, available potassium, alkali-hydrolyzable nitrogen, pH, and electrical conductivity, reflect soil water and nutrient availability as well as habitat conditions supporting stand sustainability; health risk indicators, including dead branch ratio and pest and disease incidence, reflect the stand’s ability to withstand stress; and external disturbance indicators, represented by grazing damage, reflect the degree of disturbance affecting the stand.

Based on the ecological meaning of different indicators, the indicators were classified into two types: benefit indicators and cost indicators. Higher values of benefit indicators indicate better stand health, whereas higher values of cost indicators indicate a greater degree of degradation. Table 1 presents the comprehensive health evaluation indicators for poplar shelterbelts.

### 2.4. Data Preprocessing and Standardization

To eliminate differences in the dimensions of various indicators, all indicators undergo dimensionless processing. Benefit-oriented indicators employ forward standardization:

(1) Standardization of benefit-oriented Indicators

(1) $$y_{ij}=\frac{x_{ij}-\min(x_{j})}{\max(x_{j})-\min(x_{j})}$$

where $x_{ij}$ is the raw value of the *j*th indicator at the *i*th plot; $y_{ij}$ is the normalized dimensionless value; $min(x_{j})$ and $max(x_{j})$ are the minimum and maximum values of the jth indicator across all plots, respectively; *i* = 1, 2, …, n; and *j* = 1, 2, …, m.

(2) Standardization of cost-type indicators

(2) $$y_{ij}=\frac{max(x_{j})-x_{ij}}{max(x_{j})-min(x_{j})}$$

where the symbols are defined as above.

### 2.5. Determining Indicator Weights Using the Entropy Weight Method

The entropy weight method is employed to objectively determine the weights of each indicator, following these steps:

(1) Construct the standardized matrix.

(2) Calculate the information entropy of the *j*th indicator:

(3) $$e_{j}=-k\sum_{i=1}^{n}p_{ij}\ln(p_{ij})$$

where $p_{ij}$ is the proportion of the *i*th sample plot in the *j*th indicator (derived from the standardized values), and n is the number of sample plots; and k = 1/ln(n), which is used to ensure that 0 ≤ e_j_ ≤ 1.

(3) Calculate the difference coefficient:

(4) $$g_{j}=1-e_{j}$$

where g_j_ is the difference coefficient of the jth indicator, and e_j_ is the information entropy of the jth indicator.

(4) Calculate the entropy weight:

(5) $$w_{j}=\frac{g_{j}}{\sum_{j=1}^{m}g_{j}}$$

where $w_{j}$ is the entropy weight of the jth indicator, and m is the number of evaluation indicators. The entropy weight reflects the information contribution of each indicator, effectively reducing subjective bias from manual weighting.

### 2.6. Entropy Weight–TOPSIS Model Calculation of Comprehensive Health Index

Based on the entropy-weighted indicator matrix, both positive ideal solutions (PIS) and negative ideal solutions (NIS) are constructed. The distances from sample plot i to both ideal solutions are calculated:

(6) $$D_{i}^{+}=\sqrt{\sum w_{j}(y_{ij}-y_{j}^{+}{)}^{2}},\;D_{i}^{-}=\sqrt{\sum w_{j}(y_{ij}-y_{j}^{-}{)}^{2}}$$

where $D_{i}^{+}$ and $D_{i}^{-}$ represent the distances from the ith sample plot to the positive ideal solution and negative ideal solution, respectively; w_j_ is the entropy weight of the jth indicator; y_ij_ is the standardized value of the *j*th indicator for the *i*th sample plot; and $y_{j}^{+}$ and $y_{j}^{-}$ represent the positive and negative ideal values of the *j*th indicator, respectively.

The final comprehensive health score is:

(7) $$C_{i}=\frac{D_{i}^{-}}{D_{i}^{+}+D_{i}^{-}}$$

where *C_i_* is the comprehensive health index of the *i*th sample plot. A higher *C_i_* value indicates better stand health and a lower degree of degradation.

### 2.7. Classification of Degradation Grades Based on RSR–Probit

To convert the comprehensive evaluation results into degradation grades applicable to management practice, this study first calculated the comprehensive health index (Ci) using the entropy weight–TOPSIS model. Subsequently, the RSR method combined with Probit analysis was used to classify degradation grades. First, the ranks of each plot were calculated based on the standardized indicator matrix, and the corresponding RSR values were obtained. The RSR values were then arranged in ascending order, cumulative frequencies were calculated, and the cumulative frequencies were converted into Probit values.

A linear regression model was established with RSR as the dependent variable and Probit as the independent variable. The RSR values corresponding to Probit values of 4, 5, and 6 were used to determine the classification thresholds. Finally, the degradation status of poplar shelterbelts was classified into four grades: Grade I, non-degraded; Grade II, lightly degraded; Grade III, moderately degraded; and Grade IV, severely degraded.

### 2.8. Ecological Response Characteristics of Key Indicators Among Different Degradation Grades

Because the weighting results showed that stand structure and soil conditions were the dominant first-level categories in the degradation assessment, this section focused on representative ecological response indicators closely related to these two categories, including understory community characteristics and surface soil properties. These indicators were used to interpret the ecological differences among degradation grades rather than to replace the full five-category and 18-indicator evaluation system.

To further clarify the ecological response characteristics of different degradation grades, understory community indicators and surface soil indicators were compared across Grade I–IV poplar shelterbelt plots. The analyzed indicators included understory cover, species richness index, surface soil moisture content, organic matter, alkali-hydrolyzable nitrogen, available phosphorus, and available potassium. Because some of these indicators had already been included in the comprehensive health evaluation and degradation grading system, they were not treated as completely independent external validation factors. Instead, they were used to explain the ecological response differences corresponding to different degradation grades and to provide an ecological basis for formulating grade-specific management strategies.

### 2.9. Data Processing and Statistical Analysis

**Figure 2:**
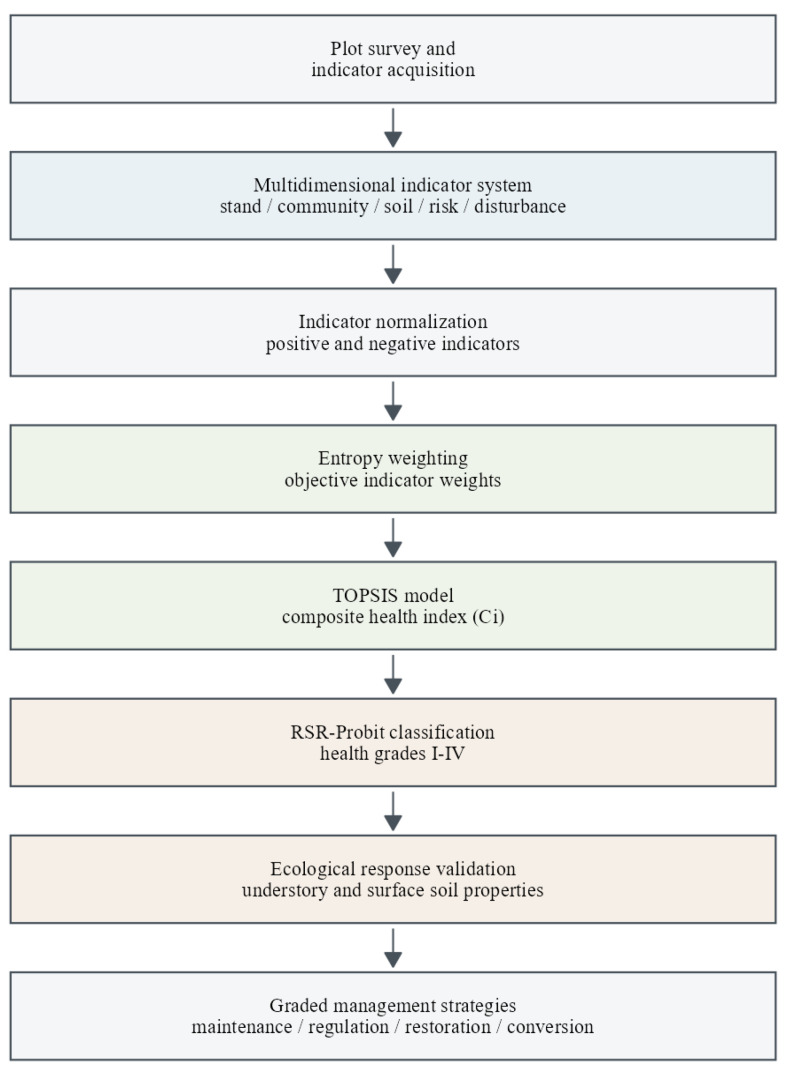
Framework for comprehensive health assessment and degradation grading of poplar shelterbelts.

Data organization and calculations were performed using R software version 4.5.2. The entropy weight method was used to calculate indicator weights, the TOPSIS model was used to calculate the comprehensive health index (Ci), and the RSR–Probit method was used to determine degradation grade thresholds (Figure 2). Differences in ecological response indicators among degradation grades were tested using one-way analysis of variance (ANOVA), followed by Tukey’s multiple comparison test. The significance level was set at *p* < 0.05. Figures were generated using R software version 4.5.2.

## 3. Results

### 3.1. Characteristics of Indicator Weight Distribution

**Figure 3:**
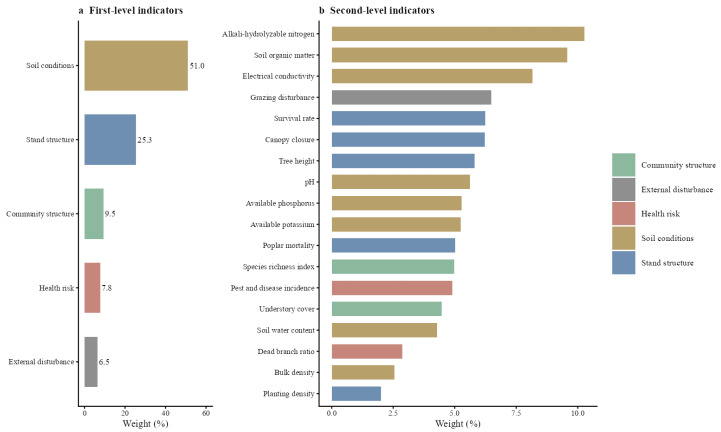
Indicator weights for health assessment and degradation grading of poplar shelterbelts in the Kubuqi Desert. (**a**) Weights of first-level indicators; (**b**) weights of second-level indicators.

The results of the entropy weight method showed that differences in the degradation status of poplar shelterbelts in the Kubuqi Desert were mainly driven by soil conditions and stand structure. Among the first-level indicators, soil conditions had the highest weight, accounting for 50.98%, followed by stand structure, with a weight of 25.30%. The weights of community structure, health risks, and external disturbances were 9.45%, 7.77%, and 6.49%, respectively (Figure 3). These results indicate that, in shelterbelts in arid and semi-arid regions, soil water and nutrient conditions are the primary factors associated with stand degradation, followed by stand structure and understory vegetation patterns. Among the second-level indicators, alkali-hydrolyzable nitrogen (10.27%), organic matter (9.57%), electrical conductivity (8.17%), grazing disturbance (6.49%), survival rate (6.25%), and canopy closure (6.22%) had relatively high weights (Table 2). This suggests that available soil nutrients, salinity status, tree survival, and external disturbances had prominent effects on the degradation assessment, whereas indicators such as bulk density and planting density had relatively low weights.

Table 2 presents the complete weighting results for all five first-level categories and 18 second-level indicators, providing a systematic overview of the contribution of each component to the health evaluation and degradation grading system.

### 3.2. Distribution Characteristics of the Comprehensive Health Index (Ci) for Poplar Shelterbelts

**Figure 4:**
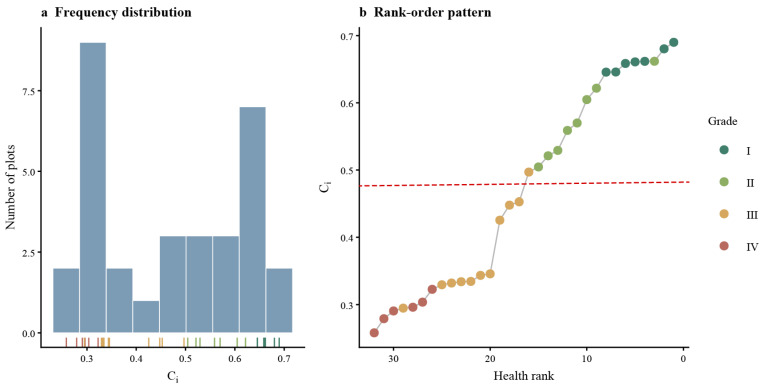
Frequency distribution and ranked distribution of the comprehensive health index (Ci) of poplar shelterbelts. (**a**) Frequency distribution of the comprehensive health index (Ci) of sampling plots and the fitted normal curve; (**b**) ranked distribution of the comprehensive health index (Ci) of sampling plots. Points represent Ci values, and the red dashed line indicates the mean value. Grade I, non-degraded; Grade II, lightly degraded; Grade III, moderately degraded; and Grade IV, severely degraded.

The entropy weight–TOPSIS method was used to calculate the distances between each plot and the positive and negative ideal solutions, thereby obtaining the comprehensive health index (Ci). A higher Ci value indicates better health status and a lower degree of degradation of poplar shelterbelts. The comprehensive health index (Ci) of the 32 plots ranged from 0.258 to 0.690, with a mean value of 0.472 and a median value of 0.475. This indicates that the overall dispersion of the health index was relatively low, with most Ci values concentrated between 0.30 and 0.60 (Figure 4a). The distribution was approximately normal-like, with only a few plots below 0.30 or above 0.60. These results suggest that the overall health status of poplar shelterbelts in the study area was at a moderate level, with relatively few plots showing extremely high or low health index values.

After the plots were ranked in descending order of Ci values, the health status of the plots showed a continuous decreasing trend (Figure 4b). The Ci values of several plots at the beginning of the ranking were slightly higher than the overall mean. This was followed by a relatively long interval in which Ci changed slowly and remained around 0.50, whereas only a few low values appeared at the end of the ranking. Overall, the distribution showed a pattern of concentration in the middle and scarcity at both extremes.

### 3.3. Classification of Degradation Grades of Poplar Shelterbelts Based on the Rank-Sum Ratio (RSR)

**Figure 5:**
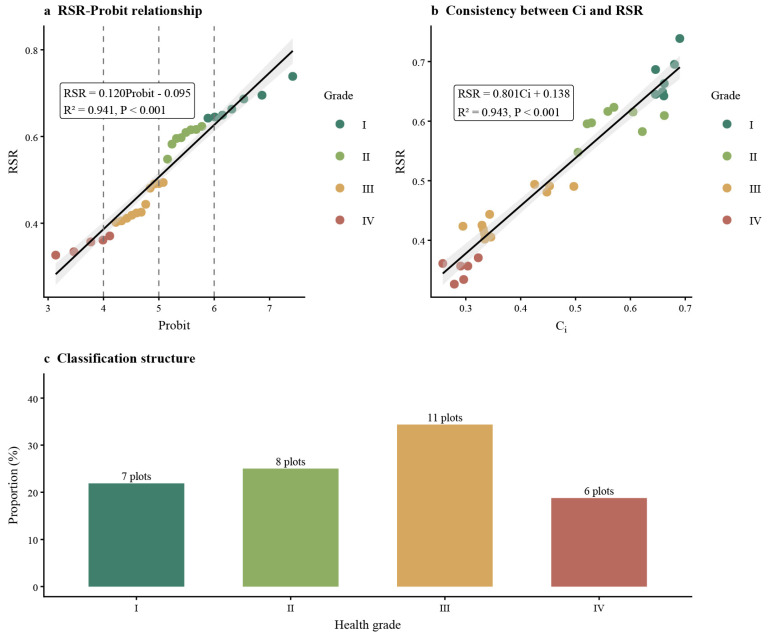
Relationships of the Rank-Sum Ratio (RSR) with Probit and the comprehensive health index (Ci) for poplar shelterbelts. (**a**) Linear relationship between the RSR and Probit; (**b**) linear relationship between the RSR and the comprehensive health index (Ci). Grade I, non-degraded; Grade II, lightly degraded; Grade III, moderately degraded; and Grade IV, severely degraded. (**c**) number and proportion of plots corresponding to each degradation grade.

Based on the comprehensive evaluation described above, the RSR method was used to rank the degradation status of the 32 plots. The RSR values were sorted in ascending order, the cumulative frequency of the mth plot was calculated, and the cumulative frequency was then converted into Probit values, which are standard normal probability units. Linear regression was performed with Probit as the independent variable and RSR as the dependent variable. The results showed a significant linear relationship between Probit and RSR (Figure 5a), and the regression equation was expressed as follows: RSR = 0.120Probit − 0.095 (R^2^ = 0.941, *p* < 0.001). The approximately linear relationship between Probit values converted from cumulative frequencies and RSR values indicates that the probability distribution characteristics can be used to classify the degradation status of poplar shelterbelts.

Probit values of 4, 5, and 6 were selected as grading nodes, corresponding to cumulative probabilities of approximately 0.16, 0.50, and 0.84, respectively. By substituting Probit values of 4, 5, and 6 into the regression equation, three corresponding RSR thresholds were obtained: 0.3862, 0.5065, and 0.6268, respectively. Based on these thresholds, the degradation status of poplar shelterbelts was classified into four grades: RSR ≥ 0.6268 was classified as Grade I (non-degraded); 0.5065 ≤ RSR < 0.6268 was classified as Grade II (lightly degraded); 0.3862 ≤ RSR < 0.5065 was classified as Grade III (moderately degraded); and RSR < 0.3862 was classified as Grade IV (severely degraded). 

Overall, Grade II and Grade III plots accounted for the highest proportions, whereas Grade I and Grade IV plots were relatively few. This indicates that most poplar shelterbelts in the study area were in the lightly to moderately degraded stages, with only a small number of plots remaining non-degraded or reaching severe degradation. This distribution pattern was consistent with the approximately normal-like distribution of the comprehensive health index (Ci), characterized by a higher concentration in the middle and fewer plots at both extremes. In addition, a significant positive correlation was observed between Ci derived from the entropy weight–TOPSIS model and RSR (Figure 5b). The grading results showed that the numbers of Grade I, Grade II, Grade III, and Grade IV plots were 7, 8, 11, and 6, respectively (Figure 5c), accounting for 21.88%, 25.00%, 34.38%, and 18.75% of the total. Among these, lightly to moderately degraded plots accounted for a relatively high proportion.

### 3.4. Relationships Between Degradation Grades of Poplar Shelterbelts and Understory Community and Soil Properties

**Figure 6:**
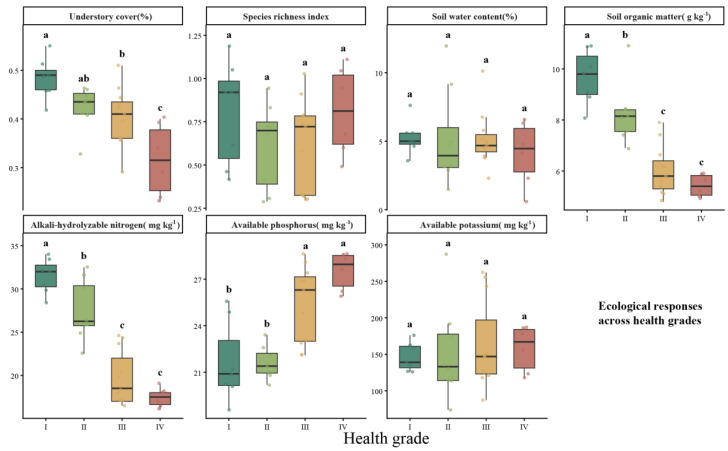
Understory community and surface soil responses among different degradation grades. Different lowercase letters indicate significant differences among degradation grades based on Tukey’s test (*p* < 0.05). Grade I, non-degraded; Grade II, lightly degraded; Grade III, moderately degraded; and Grade IV, severely degraded.

Understory community structure and surface soil properties showed varying degrees of difference among degradation grades (Figure 6). Understory cover generally decreased as the degradation grade increased from Grade I to Grade IV, decreasing from 0.48 in Grade I to 0.32 in Grade IV. Grade I was significantly higher than Grades III and IV, whereas Grade II showed an intermediate level, indicating clear differentiation in understory vegetation cover among degradation grades. Species richness index did not differ significantly among grades, and Grades I, II, III, and IV belonged to the same statistical group. This indicates that changes in degradation grade did not lead to a consistent monotonic change in understory species richness. Surface soil water content also showed no significant difference among degradation grades. Grades I, II, III, and IV belonged to the same statistical group, with mean values of 5.28%, 5.18%, 5.14%, and 4.12%, respectively, showing only a slight decreasing trend with increasing degradation severity.

The differentiation of surface soil nutrients among degradation grades was more pronounced (Figure 6). Soil organic matter and alkali-hydrolyzable nitrogen decreased continuously with increasing degradation grade. Specifically, soil organic matter decreased from 9.69 g/kg in Grade I to 5.42 g/kg in Grade IV, while alkali-hydrolyzable nitrogen decreased from 31.50 mg/kg in Grade I to 17.42 mg/kg in Grade IV. Grades I and II were significantly higher than Grades III and IV, indicating that the carbon and nitrogen supply capacity of surface soil was markedly weakened in moderately to severely degraded stands. In contrast, available phosphorus increased with increasing degradation severity, rising from 21.64 mg/kg in Grade I to 27.53 mg/kg in Grade IV. Grades III and IV were significantly higher than Grades I and II, indicating relative enrichment of soil phosphorus during degradation. Available potassium showed no significant difference among grades, and Grades I, II, III, and IV belonged to the same statistical group, with mean values ranging from 146.57 to 162.36 mg/kg. Overall, increasing degradation grade was mainly accompanied by decreased understory cover, significant declines in soil organic matter and alkali-hydrolyzable nitrogen, and a relative increase in available phosphorus, whereas species richness, surface soil water content, and available potassium showed relatively weak responses to degradation grade.

## 4. Discussion

### 4.1. Applicability of the Entropy Weight–TOPSIS–RSR Framework and the Significance of Degradation Grading

The degradation of shelterbelts in arid and semi-arid regions is typically a multidimensional process involving stand structural decline, changes in understory communities, alterations in soil conditions, and the accumulation of external disturbances. A single indicator is therefore insufficient to comprehensively reflect their degradation status. Forest health assessment emphasizes the capacity of forest ecosystems to maintain structural stability, functional integrity, and resistance to external disturbances [16,17]. In recent years, assessments of forest health and ecosystem integrity have gradually shifted from single growth indicators to comprehensive diagnosis based on multidimensional indicators related to structure, function, soil conditions, and disturbance [18,19]. Therefore, integrating multidimensional indicators into a unified evaluation framework is a necessary prerequisite for diagnosing degradation grades of poplar shelterbelts in arid sandy regions.

Compared with traditional single-factor evaluations or methods that mainly rely on subjective weighting, this study adopted a comprehensive evaluation framework combining the entropy weight method, the TOPSIS model, and the RSR method. This framework converts multidimensional indicators into a continuous health index and further classifies the evaluation results into degradation grades applicable to management practice. The entropy weight method determines indicator weights based on the dispersion of indicator data, thereby reducing bias caused by subjective weighting [25]. The TOPSIS model reflects the relative closeness of each plot to the ideal health state and is suitable for multi-indicator comprehensive evaluation [26]. The RSR method enables comprehensive ranking based on rank information and can be combined with Probit analysis to determine grading thresholds [29]. Objective weighting-based comprehensive evaluation has also been applied to assessments of desert ecosystem health, environmental governance quality, and ecological integrity [24,36,37].

In this study, RSR showed a significant linear relationship with Probit, and Ci showed a significant positive correlation with RSR, indicating that the comprehensive health index derived from the entropy weight–TOPSIS model was highly consistent with the RSR ranking results. This suggests that using RSR thresholds to classify the continuous comprehensive evaluation results into Grades I–IV is statistically reasonable. Compared with simply providing a comprehensive score, degradation grades can be more directly linked to management measures, allowing evaluation results to be transformed from plot ranking into grade-based diagnosis and management. The distribution of degradation grades showed that Grade I and Grade IV plots were relatively few, whereas Grade II and Grade III plots accounted for higher proportions. This indicates that most stands were not in extremely healthy or severely degraded conditions, but were instead in the lightly to moderately degraded stages. This pattern suggests that there remains considerable scope for management intervention in the poplar shelterbelts of the study area.

### 4.2. Degradation Information Revealed by Indicator Weights and Ecological Responses

The results of the indicator weighting analysis showed that soil conditions and stand structure were the main dimensions influencing differences in the degradation status of poplar shelterbelts in the Kubuqi Desert. Among them, soil conditions had the highest weight, indicating that topsoil nutrients and physicochemical properties made important contributions to the diagnosis of stand degradation. Ecosystem health assessment frameworks generally emphasize that ecosystem health depends not only on the biological community itself but also on the environmental foundation required to maintain structure and function [34]. Stand structure had the second highest weight, indicating that structural indicators such as survival rate, canopy closure, and tree height remain core criteria for assessing the stability of poplar shelterbelts. Previous studies on forest health assessment have also identified stand structure and soil conditions as important diagnostic dimensions [19,35].

Among the second-level indicators, alkali-hydrolyzable nitrogen, organic matter, electrical conductivity, grazing disturbance, survival rate, and canopy closure had relatively high weights. This suggests that topsoil nutrient supply, stand survival status, and external disturbances have strong indicative significance in diagnosing the degradation of poplar shelterbelts in the Kubuqi Desert. Degradation of poplar plantations in northern sandy regions is often accompanied by canopy thinning, increased mortality, and reduced protective function [9,11]. Therefore, incorporating stand structure, community structure, soil conditions, health risks, and external disturbances into a unified evaluation system helps avoid the limitations of assessing stand health based only on tree growth or a single soil indicator.

The classification of degradation grades not only reflected statistical ranking but also corresponded to ecological differences in understory communities and surface soil conditions. The results showed that understory cover differed significantly among degradation grades, indicating that understory vegetation cover responded clearly to changes in stand health status. Understory vegetation is often regarded as an important indicator of environmental change in forest ecosystems, and its growth status can reflect combined changes in canopy structure, light conditions, soil resources, and disturbance pressure [31]. Previous studies have shown that understory vegetation often responds markedly after tree mortality, canopy decline, or changes in stand structure [38,39]. Therefore, changes in understory cover can serve as important ecological signals of structural transition and community reorganization in poplar shelterbelts.

Soil nutrients showed clearer responses to degradation grades [40]. Soil organic matter and alkali-hydrolyzable nitrogen decreased significantly with increasing degradation grade, indicating that the carbon and nitrogen supply capacity of surface soil was weakened in moderately to severely degraded stands [41]. Soil organic matter is an important basis for maintaining soil structure, nutrient supply, and microbial processes. Changes in forest litter input can affect soil organic matter accumulation and nutrient cycling processes [42]. Changes in canopy structure may also alter nutrient release by affecting the microenvironment and litter decomposition processes [43]. The decline in alkali-hydrolyzable nitrogen indicates a reduced supply of plant-available nitrogen, which may limit the restoration potential of degraded stands. Poplar growth is relatively sensitive to nitrogen supply, and changes in nitrogen availability can affect its growth and carbon-nitrogen physiological processes [44].

Unlike organic matter and alkali-hydrolyzable nitrogen, available phosphorus was relatively higher in Grade III and Grade IV stands, showing a different pattern from carbon and nitrogen. This pattern may be associated with weakened tree growth, reduced phosphorus demand, or the redistribution and relative accumulation of phosphorus in surface soil during stand degradation. Phosphorus cycling differs from nitrogen cycling, and phosphorus availability is jointly regulated by multiple processes, including mineral weathering, adsorption–desorption, mineralization, and plant uptake [45]. Therefore, an increase in available phosphorus does not necessarily indicate an improvement in soil quality, but may instead reflect changes in nutrient structure in degraded stands. Considering that ecosystem responses may involve legacy effects and time-lagged processes, such changes in nutrient structure may also influence subsequent vegetation recovery [46]. Overall, increasing degradation grade was mainly accompanied by loosening stand structure, responses of understory vegetation, and declines in surface soil carbon and nitrogen, whereas the relative increase in available phosphorus may indicate a reorganization of soil nutrient structure.

### 4.3. Grade-Specific Management Strategies Based on Degradation Grades and Study Limitations

The ultimate goal of degradation assessment is not merely to obtain grading results, but to provide a basis for grade-specific management. In this study, Grades I–IV were linked to four corresponding management strategies: conservation and maintenance, density regulation, assisted restoration, and near-natural transformation. Grade I stands had relatively stable structures and favorable surface soil nutrient conditions. Therefore, management should focus on conservation, maintenance, and low-intensity monitoring, while avoiding unnecessary human disturbance. Grade II stands had already shown a certain degree of reduced structural stability, but their overall function remained acceptable. Moderate density regulation, removal of weak trees, and maintenance of stand stability should therefore be implemented to prevent further degradation. Shelterbelt management needs to consider structural stability, protective function, and long-term ecological benefits simultaneously [8]. Grade III stands were in the moderately degraded stage and represented key targets for management intervention. These stands were generally characterized by canopy thinning, clear understory vegetation responses, and decreases in soil organic matter and alkali-hydrolyzable nitrogen. Assisted restoration measures should be adopted, including adjusting understory vegetation composition, improving surface soil conditions, removing dead branches and weak trees, and supplementing drought-tolerant native shrubs according to site conditions to improve community stability. Grade IV stands had severely degraded tree-layer structures and a high degree of degradation. Continuing in situ high-density poplar replanting may make it difficult to restore the original shelterbelt structure. Therefore, Grade IV stands are more suitable for near-natural transformation, including removing dead trees and promoting the stable establishment of native herbaceous and shrub communities, so that vegetation structure can better match local resource conditions and shelterbelt functional requirements. This grade-specific management approach is consistent with state-and-transition and adaptive management theories, which emphasize that interventions with different intensities and objectives should be applied at different degradation stages [32].

It should be noted that this study focused on establishing a comprehensive health evaluation and degradation grading method for poplar shelterbelts, and used understory community and surface soil indicators to examine the ecological explanatory power of the degradation grades. However, some limitations remain. The current analysis mainly emphasized the combined effects of surface soil conditions, stand structure, and external disturbances in health evaluation. Future studies could incorporate long-term monitoring data, repeated surveys across different years, and more ecological function indicators within this evaluation framework to test the temporal stability and predictive capacity of the degradation grades. In addition, management experiments could be conducted to verify the practical effectiveness of management measures corresponding to different grades, thereby further improving the application value of this grade-based diagnostic framework.

## 5. Conclusions

Based on poplar shelterbelt plots representing different degradation levels in the Kubuqi Desert, this study showed that soil conditions and stand structure were the dominant dimensions distinguishing degradation status, indicating that site resource availability and stand structural stability are central to the diagnosis of shelterbelt degradation. The grading results showed that Grade I, Grade II, Grade III, and Grade IV stands accounted for 21.88%, 25.00%, 34.38%, and 18.75% of the plots, respectively, with lightly and moderately degraded stands representing the largest proportion. This suggests that most poplar shelterbelts in the study area were in a transitional degradation stage and still had considerable potential for management intervention. Accordingly, Grade I stands should be protected and maintained, Grade II stands should receive density regulation, Grade III stands should be managed through assisted restoration and understory vegetation adjustment, and Grade IV stands should undergo near-natural transformation. These grade-specific strategies can help align stand structure, site resource conditions, and protective function requirements, thereby supporting the renewal and rehabilitation of aging and degraded shelterbelts in arid sandy regions.

## Figures and Tables

**Table 1:** Indicators for comprehensive health evaluation and degradation grading of poplar shelterbelts.

Primary Indicator	Secondary Indicator	Indicator Type
Stand structure	Canopy closure	Positive
	Survival rate	Positive
	Poplar mortality	Negative
	Tree height	Positive
	Planting density	Negative
Community structure	Understory cover	Positive
	Species richness index	Negative
Soil conditions	Soil water content	Positive
	Bulk density	Positive
	Soil organic matter	Positive
	Available phosphorus	Positive
	Available potassium	Positive
	Alkali-hydrolyzable nitrogen	Positive
	pH	Positive
	Electrical conductivity	Positive
Health risks	Dead branch ratio	Negative
	Pest and disease incidence	Negative
External disturbance	Grazing disturbance	Negative

**Table 2:** Indicator weights for comprehensive health evaluation and degradation grading of poplar shelterbelts.

Primary Indicator	Secondary Indicator	Entropy	Utility	Weight Percent
Stand structure	Canopy closure	0.9400	0.0598	6.2276
	Survival rate	0.939	0.0600	6.2501
	Poplar mortality	0.9518	0.0481	5.0173
	Tree height	0.9441	0.0558	5.8110
	Planting density	0.9808	0.0191	1.996
Community structure	Understory cover	0.9570	0.0429	4.4723
	Species richness index	0.9521	0.0478	4.9800
Soil conditions	Soil water content	0.9588	0.0411	4.2814
	Bulk density	0.97556	0.0244	2.5436
	Soil organic matter	0.9080	0.0919	9.5710
	Available phosphorus	0.9492	0.0507	5.2863
	Available potassium	0.9496	0.0503	5.2394
	Alkali-hydrolyzable nitrogen	0.9014	0.0985	10.2665
	pH	0.9460	0.0539	5.6224
	Electrical conductivity	0.9215	0.0784	8.1697
Health risks	Dead branch ratio	0.9724	0.0275	2.8735
	Pest and disease incidence	0.9529	0.0470	4.9006
External disturbances	Grazing disturbance	0.9376	0.0623	6.4898

## Data Availability

The original contributions presented in the study are included in the article, further inquiries can be directed to the corresponding author.
